# Pharmacokinetics, safety, and tolerability of onradivir in participants with severe renal impairment and matched healthy control participants

**DOI:** 10.1128/aac.00462-25

**Published:** 2025-08-04

**Authors:** Haijun Li, Xiali Yang, Jun Zhang, Lin Chen, Mingfei Zhou, Youyun Li, Xiangxing Liu, Jiyi Huang, Jufang Huang

**Affiliations:** 1 Department of Anatomy and Neurobiology, School of Basic Medical Sciences, Central South University 618101, Changsha, China; 2 Department of Nephrology, First Affiliated Hospital of Xiamen University 117892, Xiamen, China; 3 Phase 1 Clinical Unit, The First Affiliated Hospital of Jinan University 162698, Guangzhou, China; 4 Guangdong Raynovent Biotech Co., Ltd 681093 , Guangzhou, China; Houston Methodist Hospital and Weill Cornell Medical College, Houston, Texas, USA

**Keywords:** onradivir, influenza, pharmacokinetics, renal impairment

## Abstract

Onradivir is an influenza A virus RNA polymerase basic protein 2 inhibitor that is currently under development for the treatment of influenza A. Renal impairment can influence drug absorption, metabolism, and transport, potentially altering the pharmacokinetics (PK) of onradivir. This study aimed to provide guidance on clinical dosing for patients with renal impairment by evaluating the impact of renal impairment on the PK, safety, and tolerability of onradivir in a nonrandomized, parallel, single-dose study. Participants with severe renal impairment (estimated glomerular filtration rate 15–29 mL/min) along with healthy participants (*n* = eight per group) received a single oral dose of 600 mg onradivir. All participants exhibited good safety and tolerability after oral administration of onradivir, with treatment-emergent adverse events being limited to mild or moderate severity. Compared to participants with normal renal function, the maximum plasma concentration (*C*_max_) of onradivir in those with severe renal impairment was similar; however, the area under the plasma concentration time curve from zero to the last quantifiable concentration (AUC_0*–t*_) and the AUC from zero to infinity (AUC_0–inf_) were slightly lower. The geometric mean ratios and 90% confidence intervals for *C*_max_, AUC_0–*t*_, and AUC_0–inf_ were 101.35% (63.85%–160.86%), 76.31% (52.47%–110.97%), and 76.56% (51.02%–114.90%), respectively. Severe renal impairment did not have a clinically meaningful effect on the PK, tolerability, or safety of onradivir. Therefore, no dose adjustment is necessary for patients with mild-to-severe renal impairment who are taking onradivir.

**CLINICAL TRIALS:**

This study is registered with ClinicalTrials.gov as NCT06248567.

## INTRODUCTION

Influenza is an acute viral respiratory infection that causes significant morbidity and mortality worldwide, with influenza A being the subtype most responsible for pandemics due to its high susceptibility to antigenic variation (1–3). Severe influenza primarily affects high-risk populations, including the elderly, young children, obese individuals, pregnant women, and those with chronic underlying diseases. However, it can also impact individuals in the general population (4–7). RNA polymerase is an enzyme that catalyzes the chemical reactions that synthesize RNA from a DNA template; this enzyme plays a crucial role in regulating the life cycle of the influenza A virus. Owing to the highly conserved nature of the gene sequences encoding its subunits, RNA polymerase has become a key target for modern antiviral therapies (8–10). The influenza virus RNA polymerase complex consists of three subunits: polymerase acidic protein (PA), polymerase basic protein 1 (PB1), and PBP2 (10–12). The polymerase basic protein 2 (PB2) subunit, one of the most common subunits of RNA polymerase, plays a critical role in binding to the 5′-cap domain of host pre-mRNAs, which is essential for viral mRNA translation (13–16). Onradivir (ZSP1273), developed by Guangdong Raynovent Biotech Co., Ltd., is an inhibitor of the PB2 subunit of the influenza virus RNA polymerase (17).

Preclinical studies have shown that onradivir has strong antiviral activity both *in vivo* and *in vitro* (17). Clinical trial results indicate that onradivir significantly reduces the time to alleviate symptoms in Chinese participants with influenza A (18). A complete clinical mass balance study revealed that onradivir is primarily present in plasma as the parent drug, with mean cumulative recovery rates of onradivir-related substances in urine and feces of 8.75% and 83.24% of the administered dose, respectively. Less than 1% of the parent drug was recovered in urine, whereas 59.9% was recovered in feces, suggesting that onradivir is predominantly eliminated via nonrenal pathways. The major metabolites identified in plasma, urine, and feces were glucuronide and mono-oxidized metabolites, indicating that onradivir is primarily eliminated through hepatic pathways, including glucuronidation, oxidation, and biliary excretion. Additionally, a pharmacokinetics (PK) clinical trial conducted in Chinese participants with impaired hepatic function revealed that hepatic impairment significantly affected the absorption and elimination of onradivir (19).

Given that impaired renal function can affect drug absorption, distribution, metabolism, and transport pathways in the liver and gut, particularly in patients with severe renal impairment, understanding the impact of renal impairment on the PK, tolerability, and safety of onradivir is crucial for informing appropriate dose adjustments (20–22). The aim of this study was to provide guidance regarding clinical dosing for patients with renal impairment by evaluating the impact of severe renal impairment on the PK, safety, and tolerability of onradivir in a nonrandomized, parallel, single-dose study.

## MATERIALS AND METHODS

### Study design

This was a phase I, nonrandomized, open-label, parallel, single-dose clinical trial to evaluate the PK, safety, and tolerability of onradivir in participants with normal renal function and severe renal impairment. A total of 16 participants were enrolled and assigned to either the healthy group or the renal impairment group. All participants were admitted to the clinic the day before medication administration and fasted for at least 10 h. On the following morning, all participants received a dose of 600 mg onradivir, administered with 240 mL of warm water. Participants were instructed not to consume any water for 1 h before and 2 h after administration, and food was prohibited until 4 h after administration.

### Study population

The study was conducted at the First Affiliated Hospital of Xiamen University and the First Affiliated Hospital of Jinan University in China from December 2023 to April 2024 and received approval from an independent Ethics Committee. Written informed consent was obtained from all participants (the first participant provided informed consent on 21 December 2023). This clinical trial (Clinical Trials Registration: NCT06248567; https://clinicaltrials.gov) adhered to the principles of the Declaration of Helsinki and Good Clinical Practice.

Participants aged 18–65 years, with a body weight of ≥50 kg for males and ≥40 kg for females, were eligible for this study. Additional criteria included a body mass index (BMI) of 18–28 kg/m^2^. There were no restrictions regarding ethnicity. Participants with severe renal impairment needed to meet the criteria for chronic kidney disease (CKD), indicated by markers of kidney damage or an estimated glomerular filtration rate (eGFR) <60 mL/min (determined by the CKD-EPI equation [23]) for more than 3 months and an eGFR of 15–29 mL/min (inclusive) to qualify as CKD stage IV. Participants with normal renal function were required to have an eGFR >90 mL/min and were matched with those in the severe renal impairment group based on weight (mean ± 10 kg), age (mean ± 10 years), and sex (±1 participant).

The primary exclusion criteria included females who were either pregnant or breastfeeding; individuals with uncontrolled or unstable cardiovascular diseases, those with a history of alcohol or drug abuse; and individuals who had used uridine diphosphate-glucuronosyltransferase (UGT) inhibitors or inducers. Additional exclusion criteria for the severe renal impairment group included acute renal failure, a history of kidney transplantation, or the need for dialysis during the study. For the healthy group, exclusion criteria included clinically significant abnormalities in clinical laboratory examinations or any clinically significant disease identified within 12 months prior to screening.

### PK assessment

#### Plasma

Blood samples (4 mL) were collected in K_2_-EDTA tubes at predose, 0.25 h, 0.5 h, 0.75 h, 1 h, 1.5 h, 2 h, 3 h, 4 h, 6 h, 8 h, 12 h, 16 h, 24 h, 48 h, 72 h, 96 h, and 120 h post-dose. Samples were gently inverted 8–10 times and then centrifuged at 1,700 × *g* for 10 min at 4°C to obtain clear plasma, which was separated and stored at −80°C within 1 h after collection.

The high-performance liquid chromatography-tandem mass spectroscopy (HPLC–MS/MS) assay was validated according to current guidelines for the analysis of plasma samples, with a concentration range of 4.00–10,000 ng/mL for total onradivir and 20.0–10,000 pg/mL for unbound onradivir (24). Samples were processed using protein precipitation or liquid-liquid extraction prior to HPLC-MS/MS analysis. Onradivir-d4 was used as the stable isotope-labeled internal standard (IS).

For the total onradivir assay, the mass spectrometer used was an AB Sciex Triple Quad 4500 in multiple reaction monitoring (MRM) mode, with mass transitions m/z 441.2→289.2 Da (onradivir) and m/z 445.2→293.0 Da (IS). The LC system was a Shimadzu LC-20ADXR, with an Ultimate XB C18 column (2.1 × 50.0 mm, 5.0 µm, Welch, Maryland, USA). For unbound onradivir assay, the mass spectrometer used was an AB Sciex Triple Quad 6500 in Low Mass MRM mode, with mass transitions m/z 441.2→289.2 Da (onradivir) and m/z 445.1→135.1 Da (IS). The LC system was a Shimadzu LC-20ADXR, with an ACE C18 column (2.1 × 50.0 mm, 5.0 µm, ACE, Pennsylvania, USA).

Quantification was performed by peak area ratios of onradivir vs IS, with calibration curves fitted via weighted linear regression (1/concentration^2^). The lower limit of quantitation (LLOQ) for total onradivir and unbound onradivir was 4.00 ng/mL and 20.00 pg/mL, respectively.

#### Urine

Urine samples were collected at specific time intervals: predose (omitted if the participant did not urinate), 0–6 h, 6–12 h, 12–24 h, 24–48 h, 48–72 h, 72–96 h, and 96–120 h post-dose. Urine samples were mixed and aliquoted within 1 h after being collected and then stored at −80°C within 1 h after being aliquoted.

The assay was validated according to current guidelines for analyzing urine samples in the concentration range 2.00–1,000 ng/mL, with onradivir-d4 as the stable isotope-labeled IS (24). Samples were processed using liquid-liquid extraction prior to HPLC–MS/MS analysis. The mass spectrometer used was an AB Sciex Triple Quad 4500 in MRM mode, with mass transitions *m*/*z* 441.2→289.2 Da (onradivir) and *m*/*z* 445.1→135.1 Da (IS). The LC system was the same as that used for the total onradivir assay in plasma.

Quantification was performed by peak area ratios of onradivir vs IS, with calibration curves fitted via weighted linear regression (1/concentration^2^). The LLOQ was 2.00 ng/mL.

### PK analyses

The PK parameters were calculated via a noncompartmental model with Phoenix WinNonlin version 8.4 (Certara [USA], agented by Shanghai Yuanzi Biotech Co., Ltd., Shanghai, China). Actual sampling times were used for PK calculations, with concentrations below the limit of quantification being replaced with zero before *T*_max_ (time of maximum observed concentration) and with “ND” after *T*_max_. The main PK parameters were as follows.

*C*_max_ and *T*_max_ were directly obtained from the plasma concentration-time data. The first-order rate constant (λ_*z*_) was estimated by linear regression of time vs log concentration. AUC_0–*t*_ was calculated using a linear trapezoidal method. AUC_0–inf_ was calculated as follows: AUC_0–inf_ = AUC_0–*t*_ + *C*_*t*_/λ_*z*_, where *C*_*t*_ was the last measurable concentration. The terminal elimination half-life (*t*_1/2_) was calculated as ln2/λ_*z*_. The total clearance (CL/F) was calculated as dose/AUC_0–inf_, and the distribution volume (Vz/F) was calculated as CL/λ_*z*_. The percentage of AUC_0–inf_ due to extrapolation from *T*_last_ (time of last measurable concentration) to infinity (AUC__%Extrap_) was calculated as 100 × ([AUC_0–inf_ – AUC_0–*t*_]/AUC_0–inf_). The amount of onradivir recovered from urine over the 0–120 h post-dose (Ae_0–120_), the recovery rate of onradivir in urine over the same period (Fe_0–120_), and the renal clearance (CL_r_) were calculated as follows: Ae_0–120_ = $\sum_{i=1}^{n}$ C_urine, *i*_ × V_urine, *i*_ (*i* = 1 represents the first collection interval, *n* is the total number of collection intervals); Fe_0–120_ = Ae_0–120_/dose × 100%; CL_r_/F = Ae_0–120_/AUC_0–*t*_. The fraction of unbound drug (fu) was calculated as follows: fu_*t*_ (%) = *C*_*t*_ (concentration of unbound onradivir)/*C*_*t*_ (concentration of total onradivir) × 100, and the plasma protein binding (PPB) ratio was calculated as follows: PPB_*t*_ (%) = 100 fu_*t*_ (%), where *t* represents *T*_max_ and *T*_min_ (24 h after administration).

In accordance with ICH guidelines (25), participants with an extrapolated AUC_0–inf_ percentage exceeding 20% were identified, and the corresponding AUC_0–inf_, *t*_1/2_, CL/F, Vz/F, and λ_*z*_ values were excluded from the statistical analysis. The potential impact of this exclusion on the bioequivalence evaluation (variance analysis) was assessed. In addition, participants meeting the following criteria were excluded from the PK parameter analysis: (i) serious violation of the clinical trial protocol that affects PK results or prevents PK estimation; (ii) a predose plasma concentration greater than 5% of *C*_max_; (iii) a *C*_max_ for the first sample and early samples (5–15 minutes post-dose) was not collected; (iv) vomiting within twice the median *T*_max_ in the same group; and (v) the use of concomitant medication during the trial that impacts PK parameters.

### Safety assessment

Changes in physical examination, vital sign, clinical laboratory examination (hematology, urinalysis, blood chemistry, and coagulation profile), and 12-lead electrocardiogram (ECG) signal data, along with other examination data, were surveilled. Adverse events (AEs), including severity, occurrence time, end time, duration, treatment measures, and outcomes, were also recorded.

### Statistical analysis

The sample size in this study was not determined based on statistical hypothesis testing.

Statistical analysis was conducted using SAS version 9.4 (SAS Institute Inc. [USA], agented by Beijing Jingqiang Technology Development Co., Ltd., Beijing, China). For quantitative variables, descriptive statistics included the number of participants, mean, SD, minimum, maximum, and median. For categorical variables, descriptive statistics included the frequencies and percentages.

Primary PK parameters *C*_max_ and AUC were compared between participants with renal impairment and those with normal renal function. Analysis of variance was performed on the logarithmically transformed *C*_max_ and AUC values to estimate the geometric mean ratios (GMR; renal impairment group/healthy group) and their 90% confidence interval (CI). The Wilcoxon rank-sum test was used to analyze *T*_max_, while other PK parameters were assessed using the Student’s *t*-test.

AEs were coded via the Medical Dictionary for Regulatory Activities (MedDRA version 26.0 or above) and summarized descriptively. Fisher’s exact test (two-sided) was used for the statistical analysis of adverse event incidence.

## RESULTS

### Population

A total of 16 participants (*n* = eight per group) were enrolled in this trial, all of whom were administered as planned and completed the trial. Weight, height, BMI range, and sex ratio were comparable between the renal impairment group and the healthy control group. Although the mean age of the renal impairment group was higher than that of the healthy group, the difference was within acceptable criteria. The mean eGFR was 21.63 (16.00–29.00) mL/min in the renal impairment group and 106.13 (97.00–118.00) mL/min in the healthy group (Supplementary, Table S1).

### Concentration assay

The bioanalytical assays for total onradivir in plasma, unbound onradivir in plasma, and onradivir in urine demonstrated high precision and accuracy. The maximum precision (%CV) for calibration standards and quality control samples was 6.4%, 6.6%, and 5.4% for total plasma, unbound plasma, and urinary onradivir, respectively. Mean accuracy deviations ranged from −4.1% to 3.5% for total plasma onradivir, −5.0% to 4.0% for unbound plasma onradivir, and −1.6% to 3.0% for urinary onradivir. Incurred sample reanalysis (ISR) met acceptance criteria, with pass rates of 100%, 88.9%, and 100% for total plasma, unbound plasma, and urinary onradivir assays, respectively. Several biological samples required reanalysis due to analytical issues. In the renal impairment group, one participant (A004 at 1.5 h) had a repeated total onradivir assay because the initial concentration exceeded the upper limit of quantification (ULOQ). Another participant (A005 at 24 h) underwent a repeated unbound onradivir assay due to a significant discrepancy between the initial result and the ISR value. In the healthy group, three participants (B004 at 12 h, B006 at 6 h, and B007 at 6 h) required repeated urine sample assays due to concentrations exceeding the ULOQ. These results confirm the bioanalytical method’s robust reproducibility and reliability.

### PK analysis

**Fig 1:**
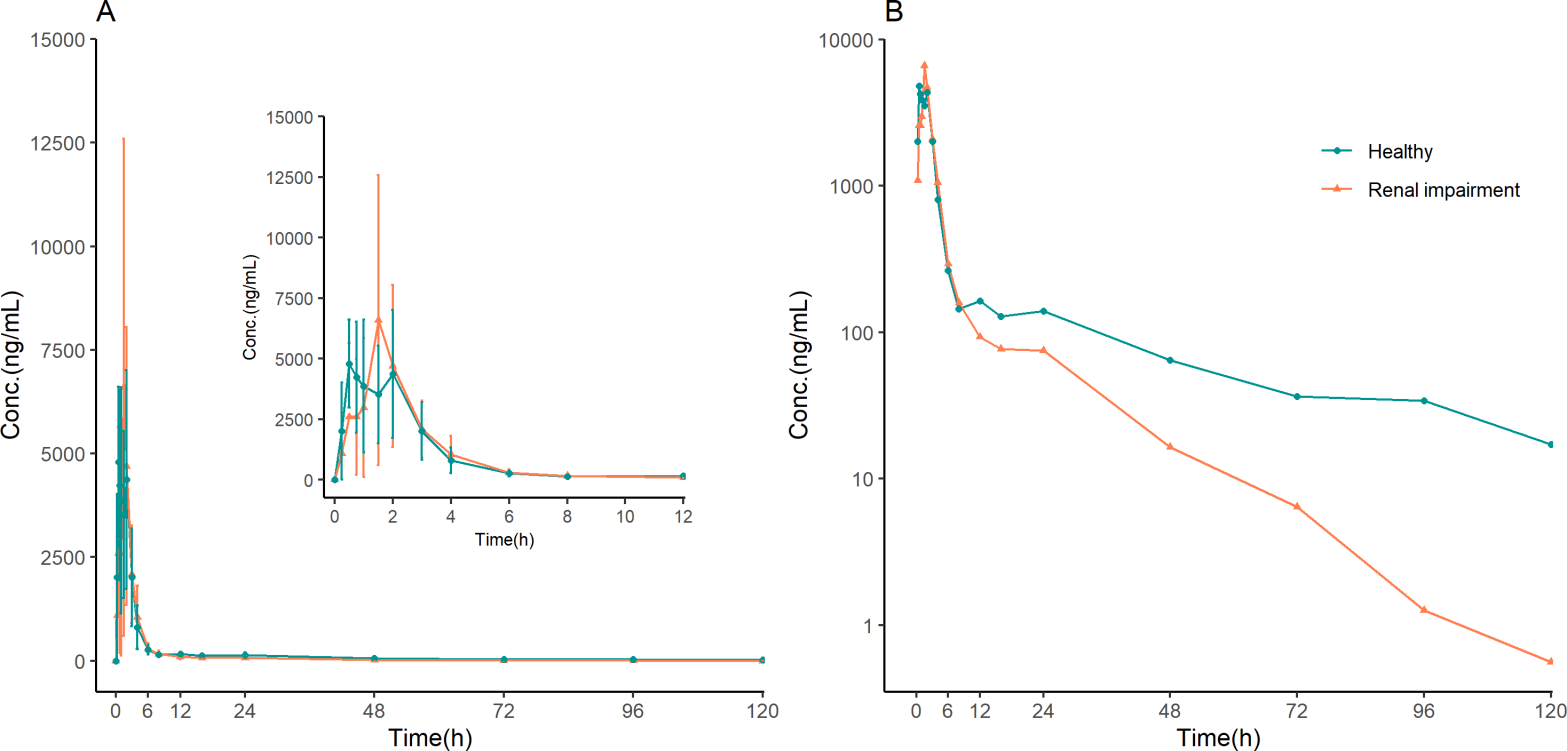
Onradivir arithmetic mean (SD) plasma concentration-time profiles on a linear scale (**A**) and arithmetic mean plasma concentration-time profiles on a semilog scale (**B**) after a single oral dose of 600 mg of onradivir to participants with renal impairment and normal renal function (*n* = 8 per group); the inset in panel A is for 0–12 h.

The mean plasma concentration-time profiles of onradivir were similar across the two groups (Fig. 1).

Following a single dose of onradivir, the median *T*_max_ was 1.48 h in the renal impairment group and 0.86 h in the healthy group, with comparable *C*_max_ values in both groups (6,848.10 ng·mL^−1^ vs 6,756.90 ng·mL^−1^, *P* > 0.05; Table 1). Compared with the healthy group, the geometric mean AUC_0–*t*_ and AUC_0–inf_ values in the renal impairment group were reduced by 23.69% and 23.44% (Table 1), respectively; however, these differences were not statistically significant (both *P* > 0.05). The renal impairment group showed a higher geometric mean elimination rate (39,178.92 mL/h vs 29,996.04 mL/h, *P* > 0.05) and shorter *t*_1/2_ (12.62 h vs 24.31 h, *P* < 0.05) compared to the healthy control group. However, renal clearance was significantly lower in the renal impairment group (3.08 mL/h vs 33.03 mL/h, *P* < 0.05).

**TABLE 1:** Summary of PK parameters in the renal impairment group and healthy group^*c*^

PK parameter	Renal impairment group (*N* = 8)	Healthy group (*N* = 8)	GMR (%)	90% CI (%)	CV (%)	*P* value
*T*_max_ (h)	1.48 (0.75, 2.95)	0.86 (0.48, 2)	/^*d*^	/	/	0.225
*C*_max_ (ng·mL^−1^)	6,848.10 (77.78)	6,756.90 (28.34)	101.35	(63.85,160.86)	56.28	0.960
AUC_0–*t*_ (ng·h·mL^−1^)	15,071.73 (55.23)	19,751.65 (31.66)	76.31	(52.47,110.97)	44.53	0.224
AUC_0–inf_ (ng·h·mL^−1^)	15,314.36 (54.95)	20,002.64 (35.27)^*a*^	76.56	(51.02,114.90)	46.56	0.265
AUC_0–inf_ (ng·h/mL)	15,314.36 (54.95)	20,881.67(34.96)^*b*^	73.34	(49.98,107.61)	45.69	0.176
λ_*z*_ (h^−1^)	0.05 (50.03)	0.03 (44.78)	/	/	/	0.008
*t*_1/2_ (h)	12.62 (50.03)	24.31 (44.78)	/	/	/	0.008
*V*_*z*_/*F* (mL)	713,087.32 (95.17)	1,052,144.9 (48.87)	/	/	/	0.173
CL/F (mL·h^−1^)	39,178.92 (54.95)	29,996.04 (35.27)	/	/	/	0.176
AUC__%Extrap_ (%)	1.11 (122.12)	1.85 (76.86)	/	/	/	0.131
Ae_0–120 h_ (ng)	46,441.44 (303.73)	652,356.02 (88.76)	/	/	/	0.001
Fe_0–120 h_ (%)	0.01 (303.73)	0.11 (88.76)	/	/	/	0.001
CL_r_/F (mL·h^−1^)	3.08 (151.63)	33.03 (92.98)	/	/	/	0.000
fu_*T*max_ (%)	0.024 (32.303)	0.013 (15.473)	/	/	/	0.000
PPB_*T*max_ (%)	99.975 (0.008)	99.987 (0.002)	/	/	/	0.001
fu_*T*min_ (%)	0.028 (46.317)	0.013 (21.348)	/	/	/	0.012
PPB_*T*min_ (%)	99.969 (0.013)	99.987 (0.002)	/	/	/	0.032

^*a*^ The AUC__% Extrap_ of B007 (healthy group) was greater than 20%, and the AUC_0–inf_ was not included in the analysis.

^*b*^ Sensitivity analysis, the AUC_0–inf_ of B007 was included.

^*c*^ The PK parameters are presented as geometric means (coefficients of variation between participants), except for *T*_max_, which is presented as median (min, max). GMR: geometric mean ratio (renal impairment group/healthy group); CI: confidence interval; CV: coefficient of variation of renal impairment group and healthy group.

^*d*^ /, not applicable.

**Fig 2:**
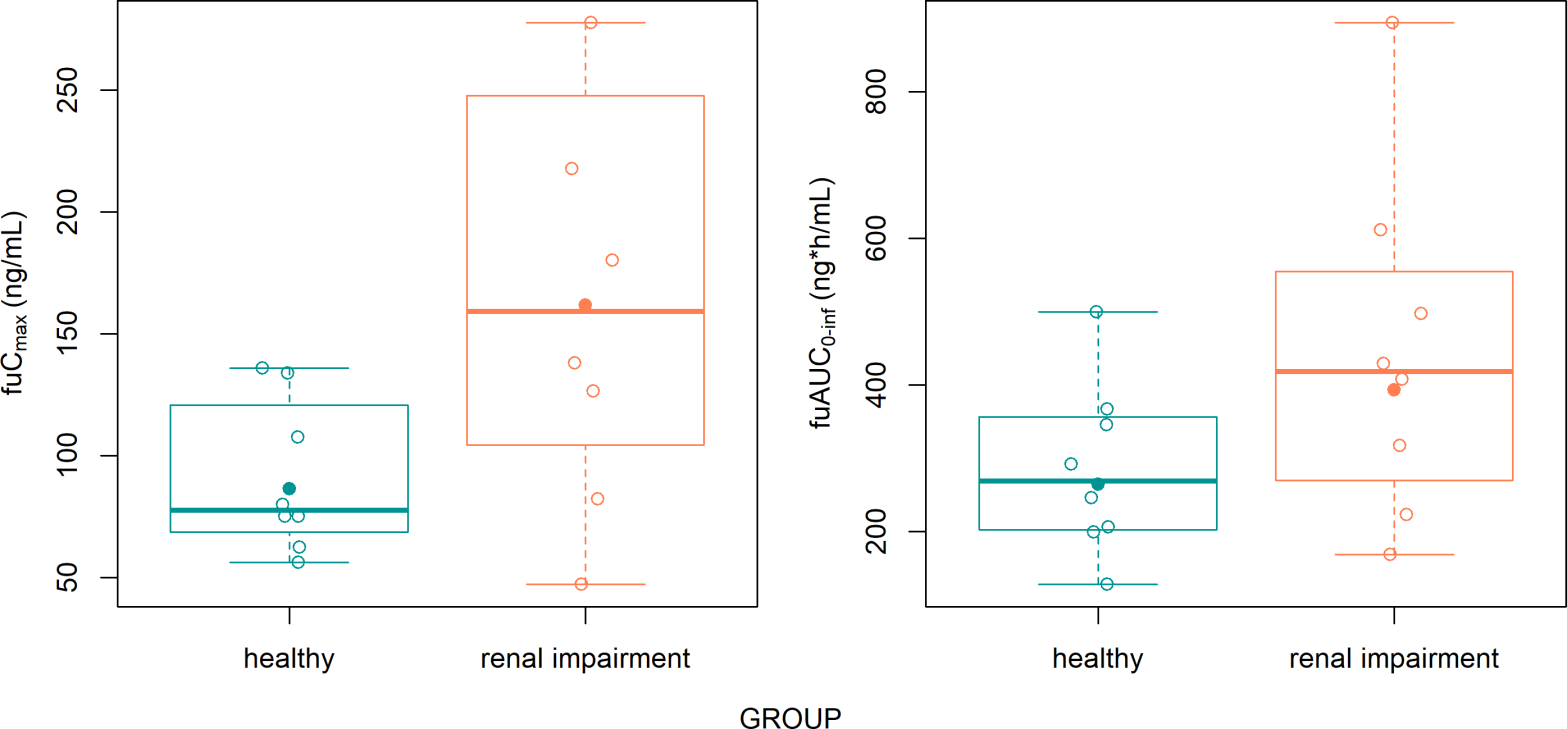
Free fraction exposure (fuC_max_ and fuAUC_0–inf_) of onradivir in the renal impairment group and healthy group. fuC_max_ = fuT_max_ × *C*_max_, fuAUC_0–inf_ = average (fu_Tmax_, fu_T24h_) × AUC_0–inf_. The solid circles represent the geometric mean, whereas the hollow circles represent individual observations. In each box, the bottom, middle, and top lines correspond to the 25th, 50th, and 75th percentiles, respectively. The lower and upper whiskers represent the minimum and maximum observations within 1.5 times the interquartile range from the box edges.

In this study, the free fraction of onradivir was measured at *T*_max_ and *T*_min_ due to its high binding affinity to plasma proteins. Both groups presented a PPB ratio above 99.9% across all participants, whereas the renal impairment group presented a greater fu. The free fraction of PK parameters (*C*_max_ and AUC_0-inf_) was estimated with fu (Fig. 2). Although differences were observed in the means and medians between the two groups, the renal impairment group presented greater variability, with most exposure (AUC_0–inf_) levels falling within the range of those of the healthy group.

In summary, there was no significant difference in exposure (*C*_max_ and AUC) between healthy participants and those with severe renal impairment.

### Safety and tolerability

Nine participants (56.3%) reported a total of 14 treatment-emergent adverse events (TEAEs), including three participants (37.5%, five TEAEs) in the renal impairment group and six participants (75.0%, nine TEAEs) in the healthy group (Table 2). The incidence of TEAEs between the two groups was not significantly different (*P* > 0.05). The TEAEs included cardiac disorders, investigations, gastrointestinal disorders, metabolism and nutrition disorders, and infections and infestations. The severity of the TEAEs ranged from grades 1 to 2, with one case of grade 2 hyperkalemia requiring drug treatment in the renal impairment group. The TEAEs were short in duration (3–8 days) and either recovered or improved before the end of the study. No participants withdrew from the study because of TEAEs.

**TABLE 2:** Incidence of TEAEs by system organ class and preferred term^*a*^

System organ class preferred term	Renal impairment group (*N* = 8)		Healthy group (*N* = 8)		*P* value (two side)
	*N* (%)	*E*	*N* (%)	*E*	
Total	3 (37.5)	5	6 (75.0)	9	0.315
Cardiac disorders	2 (25.0)	2	5 (62.5)	5	0.315
Sinus bradycardia	1 (12.5)	1	5 (62.5)	5	/^*b*^
Atrioventricular block first degree	1 (12.5)	1	0 (0)	0	/
Investigations	0 (0)	0	3 (37.5)	3	0.200
Blood triglycerides increased	0 (0)	0	1 (12.5)	1	/
Blood creatinine increased	0 (0)	0	1 (12.5)	1	/
Blood uric acid increased	0 (0)	0	1 (12.5)	1	/
Gastrointestinal disorders	1 (12.5)	1	1 (12.5)	1	1.000
Abdominal pain	0 (0)	0	1 (12.5)	1	/
Diarrhea	1 (12.5)	1	0 (0)	0	/
Metabolism and nutrition disorders	1 (12.5)	1	0 (0)	0	1.000
Hyperkalemia	1 (12.5)	1	0 (0)	0	/
Infections and infestations	1 (12.5)	1	0 (0)	0	1.000
Bronchitis	1 (12.5)	1	0 (0)	0	/

^*a*^ *N*, number of participants; *E*, number of AEs.

^*b*^ /, not applicable.

In conclusion, a single dose of 600 mg onradivir was well-tolerated and safe in both healthy participants and those with severe renal impairment.

## DISCUSSION

Onradivir, an RNA polymerase basic protein 2 inhibitor for influenza A, is primarily metabolized by UGT and eliminated via non-renal pathways, with <1% recovered in urine and 59.9% in feces. As renal impairment may alter drug PK, a phase I study assessed the PK, tolerability, and safety of a single onradivir dose in patients with severe renal impairment. The results indicated that no dose adjustment is necessary for patients with mild-to-severe renal impairment taking onradivir.

In the present study, eight participants with severe renal impairment and eight participants with normal renal function were enrolled and matched across age, sex, and weight. The safety and PK profiles of the healthy group were consistent with the findings from the phase I clinical trial (26). After a single oral dose of onradivir, good tolerability and safety were demonstrated for all participants.

The results indicated that the median *T*_max_ was numerically longer in the severe renal impairment group compared to the healthy group (1.48 h vs 0.86 h), suggesting a potential delay in absorption; however, this difference was not statistically significant and did not affect *C*_max_. However, severe renal impairment reduced onradivir exposure, with the geometric means of AUC_0–*t*_ and AUC_0–inf_ decreasing by 23.69% and 23.44%, respectively, likely due to an increased elimination rate (*t*_1/2_: 12.62 h vs 24.31 h), although these differences in AUC_0–t_ and AUC_0–inf_ were not statistically significant. Despite the increased elimination rate, cumulative urinary excretion was lower in the renal impairment group (Fe_0–120 h_: 0.01% vs 0.11%), suggesting that the reduced exposure could result from increased hepatic rather than renal clearance. Additionally, preclinical studies have demonstrated that onradivir exhibits a high PPB ratio. In this study, the free fraction of onradivir was measured at both peak and trough concentrations, and the results showed a PPB ratio exceeding 99.9% in all participants, although intergroup differences in fu were observed. While there were differences in the geomean and median values of fuAUC_0–inf_ and fuC_max_ between the groups (Fig. 2), the severe renal impairment group presented high interindividual variability, with most unbound exposure levels remaining within the range observed in the healthy group. Given the small sample size and the extremely low fu of onradivir (<0.1%), the observed differences are unlikely to have clinical significance.

A total of nine participants experienced TEAEs, including three in the renal impairment group and six in the healthy group. Most TEAEs were isolated occurrences of mild-to-moderate severity, all of which had resolved or improved by the end of the trial and lasted for a short duration. The incidence of TEAE was notably higher in the healthy group compared to the renal impairment group, which may be related to the reduced drug exposure in the latter. However, there was no statistically significant difference in the incidence of TEAE between the two groups. Given the small sample size of the present study, and considering that the incidence of TEAE in the healthy group was 12.5% (1/8) in a previously completed hepatic impairment PK study (19), the observed difference may be biased and lacks clinical significance.

In summary, compared to the healthy group, the renal impairment group exhibited a reduced geometric mean total exposure to onradivir, whereas the geometric mean unbound exposure was elevated. A comprehensive population exposure-response analysis (under submission) revealed no significant relationship between onradivir exposure (*C*_max_, AUC, or *C*_min_) and the primary efficacy endpoint (time to alleviation of symptoms) or secondary endpoints (time to temperature reduction and cessation of viral shedding). However, an association was observed between onradivir exposure (AUC and *C*_max_) and the incidence of diarrhea. These findings suggest that severe renal impairment does not have clinically significant effects on the PK of onradivir. According to Food and Drug Administration (FDA) guidance, for drugs like onradivir that are primarily eliminated via nonrenal pathways and intended for use in patients with renal impairment, if no meaningful PK differences are observed in participants with severe renal impairment, further dedicated renal impairment studies are not warranted (22). Therefore, no dose adjustment is required for patients with mild, moderate, or severe renal impairment.

A limitation of this study is the small sample size. Additionally, only a single administration of onradivir at one dose was investigated, and as such, the study does not provide insights into the potential effects of renal impairment on drug accumulation following repeated dosing or on dose linearity. This study also focused solely on the safety and PK in participants with severe renal impairment while excluding individuals with end-stage renal failure. Future studies may consider including this patient population and evaluating multiple-dose regimens across a broader dose range.

### Conclusion

Severe renal impairment participants showed good tolerance when receiving a single dose of onradivir 600 mg. No dose adjustment is necessary for patients with mild-to-severe renal impairment when taking onradivir.

## Data Availability

The original contributions from the present study are included in the article; further inquiries can be directed to the corresponding authors.

## SUPPLEMENTAL MATERIAL

Table S1 aac.00462-25-s0001.docx Participants’ demographic and baseline characteristics.

ASM does not own the copyrights to Supplemental Material that may be linked to, or accessed through, an article. The authors have granted ASM a non-exclusive, world-wide license to publish the Supplemental Material files. Please contact the corresponding author directly for reuse.

## References

[1] Taubenberger JK, Morens DM. 2010. Influenza: the once and future pandemic. Public Health Rep 125 Suppl 3:16–26. PMC2862331 20568566

[2] Iuliano AD, Roguski KM, Chang HH, Muscatello DJ, Palekar R, Tempia S, Cohen C, Gran JM, Schanzer D, Cowling BJ, et al.. 2018. Estimates of global seasonal influenza-associated respiratory mortality: a modelling study. Lancet 391:1285–1300. doi:10.1016/S0140-6736(17)33293-2 29248255 PMC5935243

[3] Gaitonde DY, Moore FC, Morgan MK. 2019. Influenza: diagnosis and treatment. Am Fam Physician 100:751–758. 31845781

[4] Fraaij PLA, Heikkinen T. 2011. Seasonal influenza: the burden of disease in children. Vaccine (Auckl) 29:7524–7528. doi:10.1016/j.vaccine.2011.08.010 21820476

[5] Molbak K, Espenhain L, Nielsen J, Tersago K, Bossuyt N, Denissov G, Baburin A, Virtanen M, Fouillet A, Sideroglou T, et al.. 2015. Excess mortality among the elderly in European countries, December 2014 to February 2015. Euro Surveill 20:21065. doi:10.2807/1560-7917.es2015.20.11.21065 25811643

[6] Zou Q, Zheng S, Wang X, Liu S, Bao J, Yu F, Wu W, Wang X, Shen B, Zhou T, Zhao Z, Wang Y, Chen R, Wang W, Ma J, Li Y, Wu X, Shen W, Xie F, Vijaykrishna D, Chen Y. 2020. Influenza A-associated severe pneumonia in hospitalized patients: risk factors and NAI treatments. Int J Infect Dis 92:208–213. doi:10.1016/j.ijid.2020.01.017 31978583

[7] Kondrich J, Rosenthal M. 2017. Influenza in children. Curr Opin Pediatr 29:297–302. doi:10.1097/MOP.0000000000000495 28346272

[8] Takashita E. 2021. Influenza polymerase inhibitors: mechanisms of action and resistance. Cold Spring Harb Perspect Med 11:a038687. doi:10.1101/cshperspect.a038687 32122918 PMC8091960

[9] Ison MG. 2015. Optimizing antiviral therapy for influenza: understanding the evidence. Expert Rev Anti Infect Ther 13:417–425. doi:10.1586/14787210.2015.1018183 25695406

[10] Peng Q, Liu Y, Peng R, Wang M, Yang W, Song H, Chen Y, Liu S, Han M, Zhang X, Wang P, Yan J, Zhang B, Qi J, Deng T, Gao GF, Shi Y. 2019. Structural insight into RNA synthesis by influenza D polymerase. Nat Microbiol 4:1750–1759. doi:10.1038/s41564-019-0487-5 31209309

[11] Reich S, Guilligay D, Pflug A, Malet H, Berger I, Crépin T, Hart D, Lunardi T, Nanao M, Ruigrok RWH, Cusack S. 2014. Structural insight into cap-snatching and RNA synthesis by influenza polymerase. Nature 516:361–366. doi:10.1038/nature14009 25409151

[12] Hengrung N, El Omari K, Serna Martin I, Vreede FT, Cusack S, Rambo RP, Vonrhein C, Bricogne G, Stuart DI, Grimes JM, Fodor E. 2015. Crystal structure of the RNA-dependent RNA polymerase from influenza C virus. Nature 527:114–117. doi:10.1038/nature15525 26503046 PMC4783868

[13] Stevaert A, Dallocchio R, Dessì A, Pala N, Rogolino D, Sechi M, Naesens L. 2013. Mutational analysis of the binding pockets of the diketo acid inhibitor L-742,001 in the influenza virus PA endonuclease. J Virol 87:10524–10538. doi:10.1128/JVI.00832-13 23824822 PMC3807387

[14] Stevaert A, Naesens L. 2016. The influenza virus polymerase complex: an update on its structure, functions, and significance for antiviral drug design. Med Res Rev 36:1127–1173. doi:10.1002/med.21401 27569399 PMC5108440

[15] Fodor E, Te Velthuis AJW. 2020. Structure and function of the influenza virus transcription and replication machinery. Cold Spring Harb Perspect Med 10:a038398. doi:10.1101/cshperspect.a038398 31871230 PMC7334866

[16] Belser JA, Maines TR, Tumpey TM, Katz JM. 2010. Influenza A virus transmission: contributing factors and clinical implications. Expert Rev Mol Med 12:e39. doi:10.1017/S1462399410001705 21144091

[17] Chen X, Ma Q, Zhao M, Yao Y, Zhang Q, Liu M, Yang Z, Deng W. 2023. Preclinical study of ZSP1273, a potent antiviral inhibitor of cap binding to the PB2 subunit of influenza A polymerase. Pharmaceuticals (Basel) 16:365. doi:10.3390/ph16030365 36986465 PMC10056986

[18] Yang Z, Li Z, Zhan Y, Lin Z, Fang Z, Xu X, Lin L, Li H, Lin Z, Kang C, et al.. 2024. Safety and efficacy of onradivir in adults with acute uncomplicated influenza A infection: a multicentre, double-blind, randomised, placebo-controlled, phase 2 trial. Lancet Infect Dis 24:535–545. doi:10.1016/S1473-3099(23)00743-0 38330975

[19] Li C, Li H, Mai J, Zhang H, Wu M, Ding Y, Huang J. 2025. Single-dose tolerability and pharmacokinetics of onradivir in Chinese patients with hepatic impairment and healthy matched controls. J Clin Pharmacol 65:226–232. doi:10.1002/jcph.6134 39287964

[20] Sun H, Frassetto L, Benet LZ. 2006. Effects of renal failure on drug transport and metabolism. Pharmacol Ther 109:1–11. doi:10.1016/j.pharmthera.2005.05.010 16085315

[21] Nolin TD, Naud J, Leblond FA, Pichette V. 2008. Emerging evidence of the impact of kidney disease on drug metabolism and transport. Clin Pharmacol Ther 83:898–903. doi:10.1038/clpt.2008.59 18388866

[22] U.S. Department of Health and Human Services. 2024. Guidance for industry, pharmacokinetics in patients with impaired renal function-study design, data analysis, and impact on dosing. Food and Drug Administration. Center for Drug Evaluation and Research(CDER)

[23] Matsushita K, Selvin E, Bash LD, Astor BC, Coresh J. 2010. Risk implications of the new CKD Epidemiology Collaboration (CKD-EPI) equation compared with the MDRD Study equation for estimated GFR: the Atherosclerosis Risk in Communities (ARIC) Study. Am J Kidney Dis 55:648–659. doi:10.1053/j.ajkd.2009.12.016 20189275 PMC2858455

[24] International Council for Harmonisation of Technical Requirements for Pharmaceuticals for Human Use (ICH). 2022. M10, Bioanalytical method validation and study sample analysis.

[25] International Council for Harmonisation of Technical Requirements for Pharmaceuticals for Human Use (ICH). 2024. M13A, Guideline on bioequivalence for immediate-release solid oral dosage forms.

[26] Hu Y, Li H, Wu M, Zhang H, Ding Y, Peng Y, Li X, Yu Z. 2021. Single and multiple dose pharmacokinetics and safety of ZSP1273, an RNA polymerase PB2 protein inhibitor of the influenza A virus: a phase 1 double-blind study in healthy subjects. Expert Opin Investig Drugs 30:1159–1167. doi:10.1080/13543784.2021.1994944 34654349

