# Supplementary material for: Pharmacokinetics, safety, and tolerability of onradivir in participants with severe renal impairment and matched healthy control participants
Source: Antimicrob Agents Chemother. 2025 Aug 4;69(9):e00462-25. doi: 10.1128/aac.00462-25 (PMC12406680; doi:10.1128/aac.00462-25)
Supplement: Table S1 — Participants’ demographic and baseline characteristics. [file aac.00462-25-s0001.docx]

**Table S1** Participants’ demographic and baseline characteristics.

| Demographic | Renal impairment group (N=8) | Healthy group function(N=8) | Overall  (N=16) |
| --- | --- | --- | --- |
| Sex (Male/Female) | 5/3 | 4/4 | 9/7 |
| Nationality (Han/Other) | 7/1 | 7/1 | 14/2 |
| Age, year | 48.50  (23.00, 60.00) | 40.13  (27.00, 46.00) | 44.31  (23.00-60.00) |
| Height, cm | 160.93  (141.00, 172.50) | 158.33  (146.40, 170.50) | 159.63  (141.00-172.50) |
| Weight, kg | 58.59  (44.00, 75.50) | 58.28  (50.50, 63.00) | 58.40  (44.00-75.50) |
| BMI^a^, kg/m^2^ | 22.45  (19.3, 25.6) | 23.28  (21.6, 25.1) | 22.86  (19.30-25.60) |
| eGFR^b^, mL/min | 21.63  (16.00, 29.00) | 106.13  (97.00, 118.00) | 63.88  (16.00,118.00) |

The data are presented as the means (min, max) or numbers.

^a^BMI: body mass index.

^b^eGFR: estimated glomerular filtration rate.
